# Supplementary material for: Alteration of the Exhaled Volatile Organic Compound Pattern in Colorectal Cancer Patients after Intentional Curative Surgery—A Prospective Pilot Study
Source: Cancers (Basel). 2023 Sep 29;15(19):4785. doi: 10.3390/cancers15194785 (PMC10571749; doi:10.3390/cancers15194785)
Supplement: Supplementary file 1 [file cancers-15-04785-s001.zip › cancers-2609738-SI.pdf]

Supplementary tables

Supplementary table S1 – Performance metrics prediction model

	Train data	Test data
**AUC**	0.79 (CI 0.68 - 0.89)	0.82 (CI 0.61 - 1.0)
**Sensitivity**	0.78 (CI 0.62 - 0.90)	0.90 (CI 0.55 - 1.0)
**Specificity**	0.73 (CI 0.56 - 0.86)	0.60 (CI 0.26 - 0.88)
**PPV**	0.74 (CI 0.62 - 0.83)	0.69 (CI 0.51 - 0.83)
**NPV**	0.77 (CI 0.64 - 0.87)	0.86 (CI 0.47 - 0.98)
**Accuracy**	76% (CI 64% - 85%)	75% (CI 51% - 91%)

Supplementary table S2 – Pre-and post-operative paired concordance 2x2 Table

	**Post-operative**		
**Pre-operative**	**Incorrect**	**Correct**	
**Incorrect**	**2**	**7**	**9**
**Correct**	**12**	**26**	**38**
	**14**	**33**	**47**
